# Ozone disrupts the communication between plants and insects in urban and suburban areas: an updated insight on plant volatiles

**DOI:** 10.1007/s11676-020-01287-4

**Published:** 2021-01-10

**Authors:** Noboru Masui, Evgenios Agathokleous, Tomoki Mochizuki, Akira Tani, Hideyuki Matsuura, Takayoshi Koike

**Affiliations:** 1 Graduate School of Agriculture, Hokkaido University, Sapporo, Japan; 2 Key Laboratory of Agrometeorology of Jiangsu Province, Institute of Ecology, School of Applied Meteorology, Nanjing University of Information Science & Technology, Nanjing, 210044 People’s Republic of China; 3 School of Food and Nutritional Sciences, University of Shizuoka, Shizuoka, Japan; 4 Research Faculty of Agriculture, Hokkaido University, Sapporo, Japan; 5 Research Center for Eco-Environmental Science, CAS, Beijing, 100085 People’s Republic of China

**Keywords:** Biological interactions, Elevated O_3_, Insect grazing, Pollination, Plant defense mechanisms

## Abstract

Plant–insect interactions are basic components of biodiversity conservation. To attain the international Sustainable Development Goals (SDGs), the interactions in urban and in suburban systems should be better understood to maintain the health of green infrastructure. The role of ground-level ozone (O_3_) as an environmental stress disrupting interaction webs is presented. Ozone mixing ratios in suburbs are usually higher than in the center of cities and may reduce photosynthetic productivity at a relatively higher degree. Consequently, carbon-based defense capacities of plants may be suppressed by elevated O_3_ more in the suburbs. However, contrary to this expectation, grazing damages by leaf beetles have been severe in some urban centers in comparison with the suburbs. To explain differences in grazing damages between urban areas and suburbs, the disruption of atmospheric communication signals by elevated O_3_ via changes in plant-regulated biogenic volatile organic compounds and long-chain fatty acids are considered. The ecological roles of plant volatiles and the effects of O_3_ from both a chemical and a biological perspective are presented. Ozone-disrupted plant volatiles should be considered to explain herbivory phenomena in urban and suburban systems.

**Supplementary information:**

The online version of this article contains supplementary material available at (10.1007/s11676-020-01287-4) to authorized users.

## Introduction

Ozone (O_3_) levels have been increasing in the last decades around the northern hemisphere, especially in east Asia (Koike et al. 2013; Akimoto et al. 2015; Feng et al. 2015, 2019a; Watanabe et al. 2017; Li et al. 2017; Nagashima et al. 2017). In general, suburban and rural areas exhibit higher average daily O_3_ levels than urban centers (Paoletti et al. 2014; Khan et al. 2017; Liu et al. 2019; Diaz et al. 2020) However, O_3_ pollution in cities has considerably increased in 2020 as a result of imposed lockdown measures due to COVID-19, suggesting potential temporal changes in the traditional differences in O_3_ concentrations between urban and suburban areas (Moser-Reischl 2019; Nakada and Urban 2020; Sharma et al. 2020; Sicard et al. 2020). Differences in O_3_ concentrations between urban and suburban areas indicate that plant–insect interactions can be affected differently.

It has been assumed that herbivory by insects is affected by physical or physiological responses of plant leaves to O_3_, which indirectly changes the lifecycle of insects, particularly under warmer climates (Duque et al. 2019). To date, research has focused on the O_3_ effect on the defensive capacity of plants, as suppression of defensive capacity leads to increased susceptibility to damage by insects. In recent research, serious insect grazing damages on avenue trees were found at relatively low O_3_ concentrations (urban centers) in northeast Asia, but such damages were not found on the same species at suburban sites with higher O_3_ levels (Takahashi et al. 2020). Plant–insect communication via biogenic volatile organic compounds (BVOCs) should be considered as a possible explanation for these observations (Agathokleous et al. 2020; Masui et al. 2020). Understanding plant–insect communications under changing atmospheric environments may help to conserve healthy forest ecosystems.

There are two roles for BVOCs: an atmospheric one and a chemical signaling one. Chemical signaling is a hub facilitating plant to plant and plant–insect communication in the environment, including in forest ecosystems (Penuelas and Llusia 2001; Heil and Bueno 2007; Trowbridge and Stoy 2013; Šimpraga et al. 2019). With regards to the atmospheric role, BVOCs contribute to O_3_ formation as O_3_ is generated from nitrogen oxides (NOx) via photochemical reactions with BVOCs under ultraviolet rays (UV) (Atkinson and Arey 2003; Kim et al. 2013; Akimoto et al. 2015). Photochemical smog, such as peroxy-acyl nitrate (PAN), is simultaneously generated thorough photochemical reaction and air pollution aggravates. VOCs act as catalytic substances in the photochemical reaction. In situations that the amount of NOx as a precursor substance of O_3_ is rapidly increasing due to industrial development, BVOCs are at the center of interest in atmospheric chemistry because global emissions of BVOCs surpass 10 times that of anthropogenic VOCs(Guenther et al. 2006; Kim et al. 2010; Im et al. 2011).

Chemical-signaling interactions via BVOCs between plants and plants/insects have biologically important roles in nature (Sharma et al. 2019). BVOCs are emitted to the atmosphere from leaves or flowers as scents (Dudareva and Pichersky 2008). There are some chemical group in these compounds; isoprene C_5_H_8_, a basic structure of BVOCs, monoterpene C_10_H_16_, sesquiterpene C_15_H_24_, while other chemically modified compounds like aldehyde or alcohol exist (Kesselmeier and Staudt 1999; Guenther et al. 2000). Although factors such as color and shape of flowers (and flower openness) contribute to their attractiveness, scent attracts insects from hundreds of meters, insects can locate their host plants by detecting unique volatile compounds or a unique blend of BVOCs (Šimpraga et al. 2016). Scent from flowers is a key factor to attract pollinators as well as herbivorous insects (Blüthgen and Klein 2011) in forest ecosystems as well (Šimpraga et al. 2019). Pollinators receive nectar and/or pollen as rewards from flowers, and pollinators and flowering plants are mutualistic (Kearns et al. 1998; Blüthgen and Klein 2011). Approximately 80–95% of flowering species in the world benefit from insect pollinators (Ollerton et al. 2011). Without pollinators, most species cannot maintain their populations and expand their gene pool by cross-fertilization; thus pollinators support the formation and sustainability of forest ecosystems (Krishnan et al. 2020).

When insects graze on leaves, they use leaf volatiles to detect target plants (Trowbridge AM and Stoy 2013). Plant–insect communication influences the productivity of a forest but in case of the grazing on avenue trees in cities the grazing damage has a negative effect on the aesthetics. A study reported that damages by herbivores are responsible for losses of net primary production up to 15% in temperate forests (Lindroth 2010). However, plants do not remain passive to be grazed upon but may affect various types of insects, not only pests but also natural enemies of the pests. Plants often have different emissions, both in quality and quantity, after being stressed by biotic and abiotic factors, for example, by drought or high temperatures (Holopainen and Gershenzon 2010) as well as by grazing (Blande et al. 2007; Copolovici et al. 2011; Takabayashi and Shiojiri 2019). The different emissions after grazing by insects, called herbivore induced plant volatiles (HIPVs), have attractant effects (Bolter et al. 1997; Sun et al. 2012; Holopainen and Blande 2013), or repellent effects to the pests (De Moraes et al. 1998, 2001; Kessler and Baldwin 2001; Kloth et al. 2012; Holopainen and Blande 2013). The attractant effect of HIPVs means that the more the pests visit and graze, the more grazing damage deteriorates at an accelerating pace. On the other hand, when HIPVs have a repellent property, the host plant avoids further grazing, which is a type of direct induced-defense system via plant volatiles. Furthermore, included in an indirect-defense system, HIPVs attract natural enemies of the pests and indirectly limit grazing damage (Shimoda et al. 2002; Ammunét et al. 2009; Klemola et al. 2012; McCormick et al. 2012).

In some cases, plants prepare a volatiles-defense system by emitting volatiles that are repellent to pests or attractant to natural enemies of the pests even before being attacked. The reason why plants can prepare to cope with pests beforehand is attributed to plant-plant communication via BVOCs. When damaged plants emit unique BVOCs, non-damaged plants respond to the emissions as an emergency alert and increase the content of defensive compounds in the leaves (Himanen et al. 2010; Girón-Calva et al. 2016; Timilsena et al. 2020). Because of this plant-plant communication, the further spread of grazing damage can be inhibited while the pests seek other undamaged host plants.

A number of researchers point out the possibility that some insecticides disrupt the activity of insects making their habitat near agricultural areas as well as harming pollinators (Christen and Fent 2017; Friedli et al. 2020). This has become a major concern for the conservation of forest ecosystems coexisting with agricultural practices. However, even if the pests or pollinators are healthy, plant–insect communication can be disrupted due to external factors affecting BVOCs such as O_3_ (Agrell et al. 2005; McFrederick et al. 2009; Fuentes et al. 2013; Šimpraga et al. 2016; Masui et al. 2020). In addition, because suburban and rural areas have relatively high O_3_ concentrations, it is important to consider the possible disruption of plant–insect communication in these areas. Therefore, the effects of O_3_ on plant–insect communication in suburban areas should be given more attention. However, information on the effect of O_3_ on communication with BVOCs is limited (Fuentes et al. 2013; Blande et al. 2014; Saunier and Blande 2019; Brosset et al. 2020). Clarifying the communication webs in elevated O_3_ sites, e.g., suburban and rural areas, can help to conserve healthy forest ecosystems under changing atmospheric environments. Furthermore, understanding the mechanism via plant volatiles will provide a perspective for other initiatives such as integrated pest management (IPM) (Vreysen 2005). In this paper, we discuss the roles of factors that regulate insect grazing activities, including defensive indicators of leaves, and focus on plant volatiles as a critical factor that may explain differences in plant–insect interactions between urban centers and suburbs.

## A parenthesis: the importance of pollinators in forests and agriculture

Pollinating insects have a significant ecological role in maintaining forest ecosystems and agroforestry. Land use systems with forest woody perennials growing among or around cultivated agricultural crops (agroforestry) can promote ecological and economical balance and secure sustainable production, helping to address sustainable development goals. In addition to their ecological importance, pollinators provide great benefits to horticultural as well as to agricultural activities. Approximately 75% of crops humans depend on the activities of pollinators to maintain their productivity (Klein et al. 2007; Eilers et al. 2011). For example, honeybees and bumblebees, possibly the most representative pollinators, are often used in greenhouse cultivation. Not only do pollinators sustain crop productivities, but they are also powerful contributors to horticultural and agricultural farming because they perfectly execute time-consuming and expensive tasks that otherwise skilled personnel would need to perform.

In modern practice, forest patches and agricultural areas often exist in the same regions, particulary in suburban and rural areas. Forest areas usually provide habitats for pollinators that contribute to agricultural crops (Rivers et al. 2018) and vice versa. Therefore, such forestry and agricultural areas complement each other by facilitating pollination (Proesmans et al. 2019). If plant-pollinator interactions are disrupted, both forest ecosystems and agricultural activities may be suppressed.

## What factor is important for insect grazing under elevated O_3_?

### O_3_ effects on physical defensive systems

Generally, leaf mass per unit area (hereafter LMA) is an indicator of physical defense to insect herbivory (Koike et al. 2006; de la Riva et al. 2016). Low LMA values mean that leaves may be easily grazed and high values that leaves may be more protected (Yamasaki and Kikuzawa 2003; Howe and Jander 2008; War et al. 2012). Elevated O_3_ levels suppress photosynthetic activities (Sitch et al. 2007; Koike et al. 2013; Watanabe et al. 2017; Grulke and Heath 2020), and photosynthesis is strongly correlated with LMA (Koike 1988; Poorter et al. 2009), meaning that elevated O_3_ can decrease LMA through physiological responses (Li et al. 2015; Shang et al. 2017).

Trichomes are also a physical defense mechanism and are specific cell constructions on the epidermal layer of leaves (e.g., hairs). They are classified into glandular and non-glandular trichomes, and provide a means for plants to defend against stresses, both abiotic (e.g., drought, freezing, UV radiation, O_3_) and biotic (e.g., pathogens, insects) (Koike et al. 2006; Hauser 2014; Oksanen 2018). For O_3_ stress, glandular trichomes reduce O_3_ uptake, while non-glandular trichomes do not have such a defensive role against O_3_ (Li et al. 2018; Oksanen 2018). With regards to herbivory, the presence of trichomes influences insect oviposition and/or feeding by various species (Vermeij 2015; Oksanen 2018). For example, trichomes interfere with insect mobility because of their morphology and also contribute to the depression effect of toxic chemicals such as phenolics that reduce the nutritional value of leaves (Matsuki et al. 2004; Schoonhoven et al. 2005; Karabourniotis et al. 2020). Both types of trichomes function as a defensive system but may not necessarily protect against the same herbivorous insect species; glandular trichomes may prevent the herbivory of an insect but may not prevent the herbivory of another which may only be prevented by a high density of non-glandular trichomes (Matsuki et al. 2004; Tian et al. 2012). Leaves under elevated O_3_ may already have a high density of glandular trichomes which act as a barrier against herbivory. Whether O_3_ induces high density glandular and/or non-glandular trichomes remains still unclear. However, there is evidence that a rapid change in glandular trichome density can occur in response to frost and to O_3_ to enhance plant tolerance, although with high variations among ecotypes of species (Prozherina et al. 2003). It was noteworthy that a shift from glandular to non-glandular trichomes was caused by increased defoliation in birch (Rautio et al. 2002).

### O_3_ effects on chemical defensive systems

Plant chemical defensive compounds such as condensed tannins, lignin, and phenolics are mostly carbon-based compounds regulated by photosynthesis (e.g. Schoonhoven et al. 2005). According to the carbon-nutrient balance (CNB) hypothesis, allocation of carbon to defensive systems becomes lower in order to keep sufficient resources for growth when plants are growing in nitrogen-rich soils and/or in conditions not favoring photosynthesis, e.g., under shade or the presence of other environmental stresses (Schoonhoven et al. 2005). Elevated O_3_ can suppress photosynthesis and lead to decreased carbon-based defense capacities, such as condensed tannins or phenolics (Matyssek et al. 2012; Sugai et al. 2020). According to the growth differentiation balance (GDB) hypothesis, some plants show a high growth rate in order to compete with other plants and to compensate for herbivorous damage, rather than allocating resources to defense when growing in an optimum environment with high availability of soil nutrients and adequate light (Herms and Mattson 1992).

**Fig. 1:**
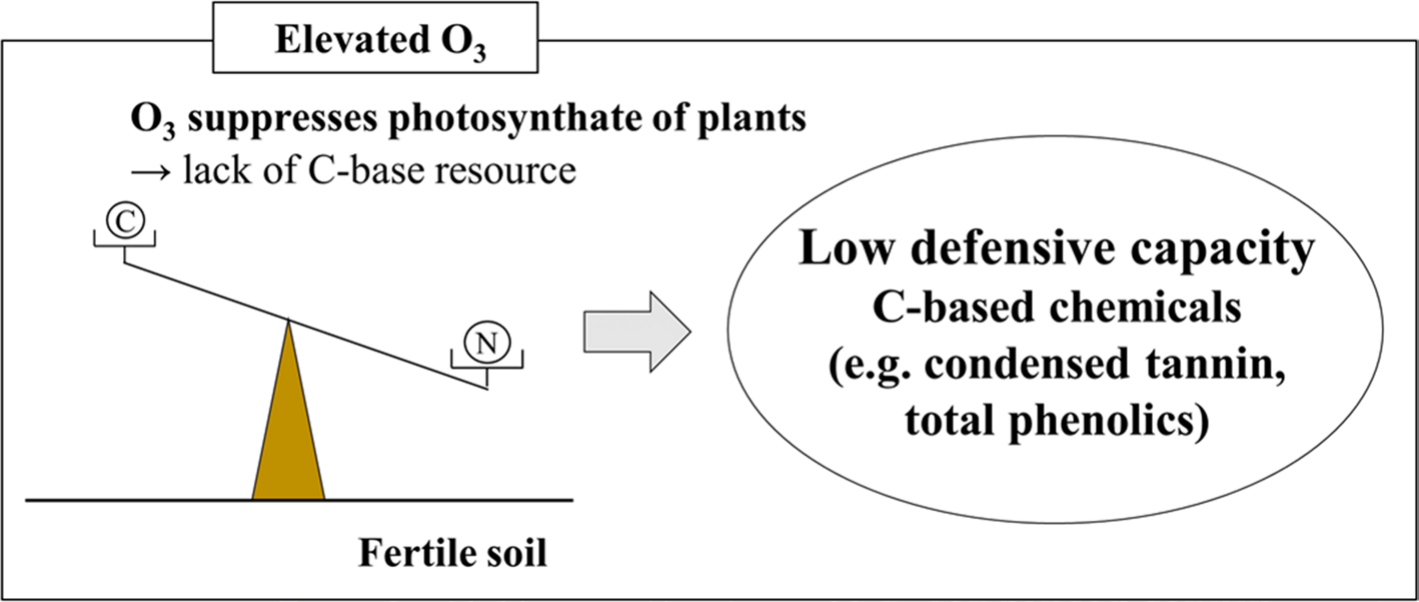
Relationship between defensive capacity of plants and environmental conditions; C is carbon and N is nitrogen (illustrated from Schoonhoven et al. 2005)

In contrast, in environments with inadequate growing conditions, plants have higher levels of defensive metabolites (Fig. 1; Schoonhoven et al. 2005; Matyssek et al. 2012; Cipollini et al. 2014). In the case of early successional species with high light demands, trees allocate their photosynthates more to growth than to defense during sapling to adult stage (Koike et al. 2006). Moreover, when trees are affected by atmospheric conditions decreasing photosynthetic efficiency such as O_3_, defensive capacities become more aggravated.

An important development in understanding the mechanism is the recognition that plant responses to O_3_ do not commonly follow a linear non-threshold model but widely follow a hormetic model (Agathokleous et al. 2019a; Bellini and De Tullio 2019; Duque et al. 2019). Ozone exposure inhibits physiological activities, however, suppression starts from a certain threshold level of exposure depending on the plant species (Agathokleous et al. 2019a). Based on the hormetic model (Agathokleous 2018, 2019a), low O_3_ concentrations, (slightly above the concentrations that plants are adapted to) can induce positive effects, including enhanced photosynthesis, and improved photosystem functioning, resulting in higher leaf areas and/or enhancement of defensive capacities by trees and other plant species. These suggest that O_3_ effects on leaf quality is not a one-way but a two-way direction.

## Limitation of traditional discussion in explaining plant–insect interactions based on foliage quality

Generally, it has been assumed that insect behavior is linked to the defensive properties of leaves (e.g. Bryant et al. 1983; Fürstenberg-Hägg et al. 2013; Agathokleous et al. 2019b). Low physical and chemical defensive properties indicate that insects can graze a plant more easily. Choice and no-choice laboratory feeding assays showed that alder leaf beetle, an oligophagous pest of alder and birch, preferred grazing elevated O_3_ leaves (over ambient O_3_ leaves) when birch leaves had low contents of condensed tannin and phenolics under elevated O_3_ (Sakikawa et al. 2016; Agathokleous et al. 2017). However, this phenomenon was not found in the field. In the field, leaf beetles grazed Japanese white birch individuals in ambient O_3_ plots more than in elevated O_3_ plots, although it would have been expected that the ozonated leaves would have been preferred based on laboratory assays (Sakikawa et al. 2016; Agathokleous et al. 2017; Abu ElEla et al. 2018). To date, much research of O_3_ effects on herbivory insects has focused on the relationship between the physiological response (photosynthesis, allocation of carbon products and nutrients) of plants and insect grazing (Manninen et al. 2000; Holton et al. 2003; Kopper and Lindroth 2003; Agrell et al. 2005; Hamilton et al. 2005; Matyssek et al. 2012; Agathokleous et al. 2017; Bubica Bustos et al. 2020). However, it is difficult to explain herbivory based on only foliage quality. Hence, apart from leaf interiors, other leaf-exterior factors potentially affected by O_3_ should be identified. For instance, plant volatiles are a major leaf-exterior factor that is now receiving increasing interest.

## New explanations: plant volatiles regulate insect grazing

### BVOCs under elevated O_3_: long-distance signals

BVOCs can attract or repel pests and pollinators, creating an important communication platform for agroforestry ecosystems (Takabayashi and Shiojiri 2019). The effect of O_3_ on BVOCs is divided into two types: (1) alteration of BVOCs emissions (an effect on plant metabolism), and (2) post-emission disruption of BVOCs in the atmosphere. In the former, BVOCs emissions can be negatively or positively affected by elevated O_3_, potentially altering the behavior of insects that detect the BVOCs and orient to space. There are some tree species that significantly change their emissions due to O_3_, while others show little or no effect, depending on the volatile compounds (Blande et al. 2007; Holopainen and Gershenzon 2010; Xu et al. 2015; Tani et al. 2017; Bison et al. 2018; Miyama et al. 2018). It should be noted that the response of BVOC emissions to elevated O_3_ is dynamic and non-linear, often biphasic (Agathokleous et al. 2018). For example, in silver birch (*Betula pendula* Roth), the emission of some volatile compounds were reduced at lower O_3_ levels but increased at higher levels (Carriero et al. 2016; Agathokleous et al. 2018). A literature meta-analysis revealed that isoprene emission is more affected by elevated O_3_ than monoterpenes (Feng et al. 2019b).

Whether BVOC emissions are affected by elevated O_3_ or not, post-emitted BVOCs in the atmosphere can be disrupted by O_3_. The lifetime of each BVOC, which ranges from a minute to hours or days, is affected by air pollutants, including O_3_ (Fuentes et al. 2000; Atkinson and Arey 2003; Arneth and Niinemets 2010). Atmospheric chemistry developments show that many volatile substances are highly reactive with O_3_. For example, the lifetime of limonene, a monoterpene, can be shorter under elevated O_3_ than one under ambient O_3_, ranging from 2 h at 26 nmol mol^−1^ O_3_ to about 40 min at 73 nmol mol^−1^ (Masui et al. 2020). The mechanism of shortened lifetime in elevated O_3_ may be explained by a structural disruption of BVOCs through an oxidizing reaction of the double-bond structure (Llusià et al. 2002; Atkinson and Arey 2003; Pinto et al. 2010). In this case, BVOCs functional role within an ecosystem is altered. If the attractant compounds show high reactivity with O_3_ (O_3_-reactive compounds), insects can be easily disoriented and wander away from their host plants in elevated O_3_ (Fuentes et al. 2013; Blande et al. 2014; Masui et al. 2020). In addition, oxidative products via reaction can show repellent effects to some insects (Glinwood et al. 2003; Mishra and Sihag 2010; Holopainen and Blande 2013), thus, the entire ecosystem may be affected by O_3_ via BVOCs communication.

Chemical analysis of plant volatiles is an important explanatory factor to consider. The evaluation of O_3_-reactive compounds is particularly necessary because there is a high possibility that these contribute to behavioral changes of insects. The composition of BVOCs depends on tree species, even in the same genus, i.e., some species emit only a few dominant compounds while others have diverse emissions, including monoterpenes (MT), sesquiterpenes (SQT), and others (Calfapietra et al. 2009; Loreto et al. 2009; Simpson and McPherson 2011). For species whose BVOCs composition has not been as yet clarified, BVOCs sampling and analysis are first needed. Moreover, heterophyllous species, ones that have leaves of more than one form on the same branch like birch (*Betula* sp.), show different physiological traits between early and late leaves (Matsuki et al. 2004; Koike et al. 2006; Agathokleous et al. 2017). Similarly, differences between early and late leaves may also be found in BVOCs emissions. BVOCs sampling has to be arranged with the phenology of herbivorous insects and heterophyllous species at the same time.

By comparing BVOCs among tree species that pests commonly graze (positive controls), compounds of high importance for the attractant property can be found. There are numerous studies that have clarified the composition of BVOCs of targeted trees but only showed the BVOCs profile, being often difficult to refer to the relationship between BVOCs and insect behavior in detail (Killiny and Jones 2017; Fancelli et al. 2018). If there is a distinct compound emission, it is easily identified and verification can proceed. However, in most cases it is assumed that the attractant property is more attributed to a combination of several compounds (Bruce et al. 2005). Thus, BVOC analyses over multiple species, including negative controls, can help to detect common BVOC combination among positive controls (BVOCs sampling and measurement is described in Supplementary Information). For example, in birch, whose main pest is the alder leaf beetle, Japanese white birch (*Betula platyphylla* var. *japonica* Hara) and alder (*Alnus japonica* (Thunb.) Steud) are positive controls; Japanese rowan (*Sorbus commixta* Hedl) and Korean mulberry (*Morus australis* Poiret) are negative controls that grow in the same area and time with positive controls in Hokkaido, Japan (Masui et al. unpublished).

### Long-chain fatty acids under elevated O_3_; short-distance signals

Long-chain fatty acids (LCFAs) and their composition can also regulate the behavior of insects via oxidation by elevated O_3_ (Manosalva et al. 2011). LCFAs are not included in BVOCs but they function as signal chemicals. In previous studies, female adult beetles of *Hylastinus obscurus* Marsh. (Coleoptera: Chrysomelidae) were attracted to LCFAs lauric, palmitic and oleic fatty acids, and red pumpkin beetle (*Aulacophora foveicollis* Lucas) to myristic, palmitoleic, α-linolenic, and nonadecanoic acids (Mukherjee and Barik 2014). Females are assumed to be more attracted to olfactory signals than males because they have to detect host plants for oviposition as well as for feeding (Mukherjee and Barik 2014). Because of the low volatility of LCFAs, insects sense them from shorter distances (Manosalva et al. 2011) compared with BVOCs. Thus, it is possible that LCFAs enable insects to detect host plants after being attracted by BVOCs from a long distance. In addition, oviposition of insects can be influenced by LCFAs on the surface of the oviposition site such as seeds or leaves (Parr et al. 1998; Li and Ishikawa 2006). Although it is still unknown how LCFAs react with O_3_ in the atmosphere as BVOCs do, the amount of LCFAs in the tissue of leaves can be decreased by O_3_ uptake, which means decreased olfactory information for insects to detect.

Comparisons between pre- and post-exposure to O_3_ based on a GC/MS analysis, shows the dynamics of LCFA composition in elevated O_3_. Previously, the relationship was studied by evaluating malondialdehyde (MDA), an indicator of lipid peroxidation (Calatayud et al. 2003; Cassimiro and Moraes 2016). However, the actual changes to compounds by O_3_ have not, as yet, been well researched. Furthermore, the effect of long-term O_3_ exposure to plants in open-field experiments remains unknown. For example, the GC/MS for major LCFAs, such as palmitic acid (C_16:0_), linolenic acid (C_18:3_), linoleic acid (C_18:2_), oleic acid (C_18:1_) and stearic acid (C_18:0_) can be relatively easily analyzed with standard samples.

### Olfactory response test

Several studies have examined whether elevated O_3_ exposure can disrupt plant–insect communication (Fuentes et al. 2016; Girón-Calva et al. 2016; Agathokleous et al. 2017; Mofikoya et al. 2018; Sugai et al. 2020). These studies support the observation that the degradation of BVOCs by elevated O_3_ is a key driver of the disruption. To support the results in the field and to identify the attractant or repellent property of each BVOC, olfactory response tests are needed. One of the tests is electroantennography (EAG), which enables the detection of whether each compound is active on the insect’s antennae (Bruce et al. 2005; Fernandez and Hilker 2007; Feng et al. 2017; Germinara et al. 2019; Iovinella et al. 2020). EAG analysis is a remarkable and useful technique for olfactory experiments; however, it does not indicate whether the insect is attracted or repelled by a compound and does not show the function of BVOCs, including all volatile compounds from plants. The Y-tube olfactometer, (Y-tube preference test), is a simpler and more effective method in vitro. In this test, air A (with BVOCs) flowing from a side of an arm and air B (BVOCs mixed with O_3_) from the other arm at the same flow rate, an insect has the task to move from the mouth of Y-shaped glass tube and select one of the two arms (Air A or Air B) to visit (Takabayashi and Dicke 1992; Shimoda et al. 1997; 2002; Brilli et al. 2009; Fuentes et al. 2013; Mukherjee et al. 2014; Masui et al. 2020). Attractant properties of BVOCs can be demonstrated by simultaneously comparing two conditions, e.g. an ambient O_3_ level vs an elevated O_3_ level that is artificially created (Pinto et al. 2007a, b). The Y-tube test can provide clear results of BVOCs as phenomenon in close-to-reality simulation.

## Plant–insect communication through plant volatiles under elevated O_3_

**Fig. 2:**
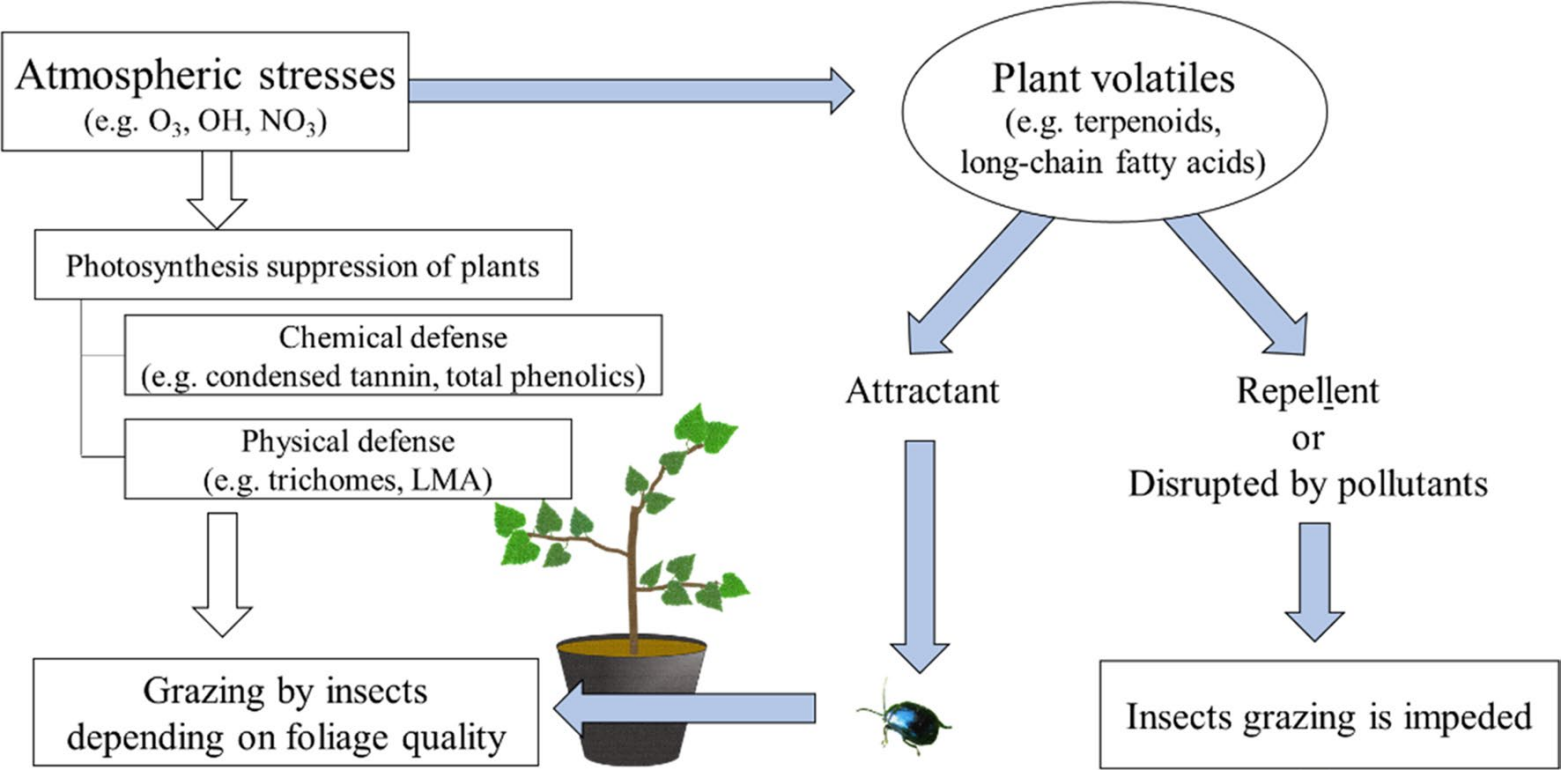
A new insight into the behavior of insects with plant volatiles under elevated O_3_. White arrows indicate traditional discussion for herbivory explained with foliage qualities such as defensive capacities. The processes indicated by white arrows mean an explanation with traditional discussion and blue arrows mean new processes with plant volatiles, incorporated to traditional discussion as shown in main text

If olfactory cues show an attractant property, insects can visit and foliage quality secondarily will affect the feeding insects (Fig. 2). In contrast, if O_3_ disrupts BVOC signals from plants to insects, or the BVOCs act as a repellent, insects may not be able to visit and thus, the chemical and physical defense of leaves do not play any role in plant–insect interaction, regardless of the actual quality. Therefore, in addition to the traditional insight of leaves as a feeding source, the effect of O_3_ on plant volatiles as olfactory cues should be taken into consideration when plant herbivory under elevated O_3_ is studied in the future. The Y-tube preference test, as a biological assay, can show the role of plant volatiles (Fuentes et al. 2013; Masui et al. 2020) and the changes under polluted air. Furthermore, by chemical analysis (e.g., using GC/MS) to identify important profiles for attractants of BVOCs, a better understanding of the interaction between O_3_ and plant volatiles can be achieved.

## Conclusions and perspectives

The considerable temporary increase in O_3_ pollution in cities worldwide subjected to “lockdown” against the spreading of the severe acute respiratory syndrome coronavirus 2 (SARS-CoV-2) (Nakada and Urban 2020; Sharma et al. 2020; Sicard et al. 2020) suggests that plant–insect interactions in urban and suburban agroforestry systems may be threatened in a shorter term due to rapid changes in anthropogenic activities.

Most studies on plant–insect interactions under O_3_ have been carried out in urban and suburban areas. This creates an important knowledge gap of O_3_ effects on plant–insect communication in remote/rural and mountainous natural forests, and especially at high altitudes, considering that O_3_ concentrations tend to increase with increasing altitude and that high altitudes have considerably higher O_3_ levels than low altitudes (Schultz et al. 2017; Saitanis et al. 2020). Hence, studies of how O_3_ affects plant–insect interaction at high altitudes are also needed.

Recently considerable research of O_3_ effects on plant volatiles have been carried out on agricultural crops (Fuentes et al. 2013; Farré-Armengol et al. 2016; Khaling et al. 2016, 2020; Acton et al. 2018; Mofikoya et al. 2018; Agathokleous et al. 2019b; Duque et al. 2019). Although there are some recent studies on O_3_ effects on forest tree volatiles (Xu et al. 2015, 2019; Yuan et al. 2020), the studies remain much fewer relatively to crops. This lack of studies with trees may be attributed to the practical difficulty in conducting such experiments with trees (in O_3_-FACE systems) in terms of time, effort and resources needed. New studies of plant volatiles with forest and avenue trees should be conducted to conserve healthy forest ecosystems in different environments facing the threat of elevated O_3_.

In conclusion, biological communication via plant volatiles (biogenic volatile organic compounds, long-chain fatty acids) between plants and insects in urban and suburban areas should be of concern. Traditional discussions were based on foliage quality (leaf mass/unit area, condensed tannin, phenolics, lignin, nitrogen content), which was rightly assumed to directly affect pests, pollinators, and natural enemies. However, a new insight based on communication between plants and insects should be also considered. Plant volatiles as olfactory cues can be altered by air pollutants such as O_3_ in the atmosphere, thereby altering the activity of insects before they arrive at the leaves and are affected by the chemical and physical foliage quality. This review serves as the basis to encourage further studies based on this insight centered on plant–insect communication via plant volatiles.

## Supplementary information

Below is the link to the electronic supplementary material.

Supplementary material 1 (DOCX 18 KB)
