# Supplementary material for: Ozone disrupts the communication between plants and insects in urban and suburban areas: an updated insight on plant volatiles
Source: J For Res (Harbin). 2021 Jan 10;32(4):1337–49. doi: 10.1007/s11676-020-01287-4 (PMC7797194; doi:10.1007/s11676-020-01287-4)
Supplement: Supplementary file 1 — Supplementary material 1 (DOCX 18 KB) [file 11676_2020_1287_MOESM1_ESM.docx]

**Supplementary information**

**Ozone disrupts the communication between plants and insects in urban and suburban areas: An updated insight focusing on plant volatiles**

**Noboru Masui^1^ 🞄 Evgenios Agathokleous^2^ 🞄 Tomoki Mochizuki^3^ 🞄 Akira Tani^3^ 🞄 Hideyuki Matsuura^4^ 🞄 Takayoshi** **Koike**^4,5^

^1^ Graduate School of Agriculture, Hokkaido University, Sapporo, Japan; nmasui_agr@frontier.hokudai.ac.jp

^2^ Key Laboratory of Agrometeorology of Jiangsu Province, Institute of Ecology, School of Applied Meteorology, Nanjing University of Information Science & Technology (NUIST), Nanjing 210044, P. R. China; evgenios@nuist.edu.cn

^3^ School of Food and Nutritional Sciences, University of Shizuoka, Shizuoka, Japan; tomokimochizuki1224@yahoo.co.jp, atani@u-shizuoka-ken.ac.jp,

^4^ Research Faculty of Agriculture, Hokkaido University, Sapporo, Japan; matsuura@chem.agr.hokudai.ac.jp, tkoike@for.agr.hokudai.ac.jp

^5^ Research Center for Eco-Environmental Science, CAS, Beijing 100085, P. R. China

**Sampling and measurement of BVOCs**

The leaf cuvette method (Tani and Kawawata 2008; Mochizuki et al. 2017) or the Branch Chamber method (Tani and Kawawata 2008; Masui et al. 2020) are adopted for BVOCs sampling and measurement by gas chromatography. The leaf cuvette method can be used for leaf-level sampling, using a portable gas exchange measurement system (e.g. LiCor LI-6400). In the LI-6400, the air stream from the cuvette originally flow back into a built-in infrared gas analyzer (IRGA) in the cuvette. In the leaf cuvette method, additionally, air stream from the cuvette is supplied into sampling port where sampling Teflon tube is attached, as well as into the IRGA. This method has the merit that can always collect target compounds at fixed environmental factors (e.g. light intensity, leaf temperature, CO_2_ concentration). However, this method is suitable for sampling target compounds from broad leaves, but is hard to obtain the representative temperature of the conifer needles, although sampling can be conducted.

For tree branches and small individual plants, branch chamber method can be used, enabling to obtain data at a more realistic scale. A sample branch (or individuals) is enclosed in a transparent fluorinated ethylene-propylene copolymer (FEP) bag. Purified air (e.g. through charcoal filter) flows into the bag from an inlet port on the bag at constant flow rate. Then, air stream flows out from the sampling port at the opposite side of inlet port, Teflon tube attached on outside of sampling port adsorb target compounds. The problem of fluctuating environmental factors can be kept minimal by sampling in a controlled room at constant temperature and light condition. In this method, care should be exercised to keep the air flow rate high so that water vapor condensing can be avoided in the bag, because high water vapor in the bag causes loss of sampling target compounds and leads to underestimating the measured amounts.

**References**

Masui N, Mochizuki T, Tani A, Matsuura H, Agathokleous E, Watanabe T, and Koike T (2020) Does ozone alter the attractiveness of japanese white birch leaves to the leaf beetle *Agelastica coerulea* via changes in biogenic volatile organic compounds (BVOCs): An examination with the Y-tube test. Forests 11: 58. https://doi.org/10.3390/f11010058.

Mochizuki T, Watanabe M, Koike T, and Tani A (2017) Monoterpene emissions from needles of hybrid larch F1(*Larix gmelinii* var. *japonica* × *Larix kaempferi*) grown under elevated carbon dioxide and ozone. Atmos Environ 148:197–202. https://doi.org/10.1016/j.atmosenv.2016.10.041.

Tani A, and Kawawata Y (2008) Isoprene emission from the major native Quercus spp. in Japan. Atmos Environ 42(19):4540–4550. https://doi.org/10.1016/j.atmosenv.2008.01.059.
